# Correction: Briggs, D.J.; Moore, S.M. The Route of Administration of Rabies Vaccines: Comparing the Data. *Viruses* 2021, *13*, 1252

**DOI:** 10.3390/v14071368

**Published:** 2022-06-23

**Authors:** Deborah J. Briggs, Susan M. Moore

**Affiliations:** Department of Diagnostic Medicine/Pathobiology, College of Veterinary Medicine, Kansas State University, Manhattan, KS 66506, USA; smoore@vet.ksu.edu

The authors wish to make the following corrections to this paper [1]:

## Correction to the Second Paragraph in Section 2.2. Efficacy

Administering CCVs by the ID route also required proof of safety and effectiveness. One of the first clinical studies to investigate the efficacy of ID PEP was conducted in Thailand by Warrell et al. and included 0.1 mL doses of HDCV administered at eight sites on Day 0, four sites on Day 7, one site on each of Days 21 and 91 [29]. All patients survived exposure to confirmed rabid animals. This study and the study also included serological evidence of immunogenicity. Further clinical trials conducted in Thailand examined reduced ID PEP regimens in patients exposed to suspect and later confirmed rabid animals [30,31]. The clinical trial conducted by Chutivongse et al. enrolled 100 patients that had been severely bitten by confirmed rabid animals and all patients were followed for one year after vaccination. All patients were confirmed to be alive one year after the final dose of vaccine was administered. These initial studies provided the proof needed that ID PEP was as effective as IM PEP when administered according to the schedules administered in the clinical trials and have served as models for designing additional clinical trials evaluating the effectiveness of new PEP regimens.

## Reference 29

Warrell, M.J.; Nicholson, K.G.; Warrell, D.A.; Suntharasamai, P.; Chanthavanich, P.; Viravan, C.; Sinhaseni, A.; Chiewbambroongkiat, M.K.; Pouradier-Duteil, X.; Xueref, C.; et al. Economical multiple-site intradermal immunisation with human diploid-cell-strain vaccine is effective for post-exposure rabies prophylaxis. *Lancet* **1985**, *1*, 1059–1062. http://doi.org/10.1016/s0140-6736(85)92367-0. 

## Reference 31

Phanuphak, P.; Khawplod, P.; Sirivichayakul, S.; Siriprasomsub, W.; Ubol, S.; Thaweepathomwat, M. Humoral and Cell-mediated Immune Responses to Various Economical Regimens of Purified Vero Cell Rabies Vaccine. *Asian Pac. J. Allergy Immunol.* **1987**, *5*, 33–37.
